# An Expanded Analysis of Pharmacogenetics Determinants of Efavirenz Response that Includes 3′-UTR Single Nucleotide Polymorphisms among Black South African HIV/AIDS Patients

**DOI:** 10.3389/fgene.2015.00356

**Published:** 2016-01-07

**Authors:** Marelize Swart, Jonathan Evans, Michelle Skelton, Sandra Castel, Lubbe Wiesner, Peter J. Smith, Collet Dandara

**Affiliations:** ^1^Division of Human Genetics, Department of Pathology and Institute of Infectious Disease and Molecular Medicine, Faculty of Health Sciences, University of Cape TownCape Town, South Africa; ^2^Division of Clinical Pharmacology, Faculty of Health Sciences, University of Cape TownCape Town, South Africa

**Keywords:** 3′-UTR, efavirenz, HIV/AIDS, pharmacogenetics, sensitivity, single nucleotide polymorphism, specificity, South Africa

## Abstract

**Introduction:** Efavirenz (EFV) is a non-nucleoside reverse transcriptase inhibitor prescribed as part of first-line highly active antiretroviral therapy (HAART) in South Africa. Despite administration of fixed doses of EFV, inter-individual variability in plasma concentrations has been reported. Poor treatment outcomes such as development of adverse drug reactions or treatment failure have been linked to EFV plasma concentrations outside the therapeutic range (1–4 μg/mL) in some studies. The drug metabolizing enzyme (DME), CYP2B6, is primarily responsible for EFV metabolism with minor contributions by CYP1A2, CYP2A6, CYP3A4, CYP3A5, and UGT2B7. DME coding genes are also regulated by microRNAs through targeting the 3′-untranslated region. Expanded analysis of 30 single nucleotide polymorphisms (SNPs), including those in the 3′-UTR, was performed to identify pharmacogenetics determinants of EFV plasma concentrations in addition to *CYP2B6 c.516G*>*T* and *c.983T*>*C* SNPs.

**Methods:** SNPs in *CYP1A2, CYP2B6, UGT2B7*, and *NR1I2* (PXR) were selected for genotyping among 222 Bantu-speaking South African HIV-infected patients receiving EFV-containing HAART. This study is a continuation of earlier pharmacogenetics studies emphasizing the role of genetic variation in the 3′-UTR of genes which products are either pharmacokinetic or pharmacodynamic targets of EFV.

**Results:** Despite evaluating thirty SNPs, *CYP2B6 c.516G*>*T* and *c.983T*>*C* SNPs remain the most prominent predictors of EFV plasma concentration.

**Conclusion:** We have shown that *CYP2B6 c.516G*>*T* and *c.983T*>*C* SNPs are the most important predictors of EFV plasma concentration after taking into account all other SNPs, including genetic variation in the 3′-UTR, and variables affecting EFV metabolism.

## Introduction

Efavirenz (EFV) is a non-nucleoside reverse transcriptase inhibitor (NNRTI) used as part of highly active antiretroviral therapy (HAART) in the treatment of human immunodeficiency virus/acquired immune deficiency syndrome (HIV/AIDS). Approximately 70% of the 2.5 million HIV-infected South Africans on HAART as part of first-line antiretroviral therapy (ART) is now given EFV once-daily (600 mg) in the following combination; tenofovir disoproxil fumarate (TDF) + emtricitabine (FTC) or lamivudine (3TC) + EFV (UNAIDS, 2013). EFV has a half-life of 40–55 h and is preferred because of its effective virologic suppression (Raffi et al., 2014). Multiple large randomized controlled trials and cohort studies have shown potent EFV-related viral suppression with many patients having viral RNA levels < 50 copies/mL for up to 6 years of follow-up (Gulick et al., 2006; Cassetti et al., 2007).

Despite its efficacy and virologic potency, EFV exposure has been linked to development of serious central nervous system (CNS) side effects in 20–74% of patients (Fumaz et al., 2002; Hawkins et al., 2005; Abah et al., 2015). The side effects include dizziness, headaches, depression, nightmares, and insomnia (Adkins and Noble, 1998; Marzolini et al., 2001). Abah et al. (2015) reported an incidence rate of 40.5 adverse drug reactions (ADRs) per 1000 person-years on EFV-based treatment among Nigerian HIV/AIDS patients, while up to 74% of HIV/AIDS patients from Uganda reportedly experienced at least one EFV-related neuropsychiatric symptom within 12 weeks of therapy (Mukonzo et al., 2013). Nearly 43% of Zimbabwean HIV/AIDS patients reported CNS toxicity after initiation of EFV-containing HAART (Dhoro et al., 2015). EFV associated symptoms range from mild to moderate in severity and subside progressively within a month after treatment initiation (Moyle, 1999a,b). The severity or persistence of adverse effects can result in EFV discontinuation and has been reported in about 4–50% of patients (Marzolini et al., 2001; Ward and Curtin, 2006; Kenedi and Goforth, 2011; Leutscher et al., 2013). EFV concentrations in plasma have been shown to be predictive of treatment outcomes in certain studies, with low concentrations potentially resulting in treatment failure, while CNS toxicity being observed three-times more frequent among patients with high EFV plasma concentrations (Marzolini et al., 2001; Kenedi and Goforth, 2011; Leutscher et al., 2013). Achieving the correct EFV dosage and plasma concentrations within the suggested therapeutic range of 1–4 μg/mL is, thus, crucial. This observation led to research focusing on identifying genetic causes of slow or fast EFV metabolism and risk factors of CNS toxicity.

EFV is metabolized to 8-hydroxy-EFV (major metabolite) primarily by cytochrome P450 (CYP) 2B6 and to a minor extent by CYP3A. 7-hydroxy-EFV and N-glucuronide-EFV metabolites are formed by CYP2A6 and UDP glucoronosyltransferases (UGT) 2B7, respectively (Ward et al., 2003; Arab-Alameddine et al., 2009; di Iulio et al., 2009). The formation rate of both 8-hydroxy-EFV and N-glucuronide-EFV is variable between human microsomal samples and points to the involvement of genetic variation in genes coding for drug metabolizing enzymes (DMEs) (Ogburn et al., 2010; Bae et al., 2011).

*CYP2B6* genetic variants, specifically *rs3745274 (c.516G*>*T)* and *rs28399499 (c.983T*>*C)*, have been investigated in relation to EFV pharmacokinetics. Numerous studies have reported an association between the *CYP2B6 c.516T*-allele and increased EFV plasma concentrations, reduced clearance, or increased drug exposure (Wyen et al., 2008; Holzinger et al., 2012; Cortes et al., 2013; Swart et al., 2013; Sinxadi et al., 2015). Patients with the *CYP2B6 c.516G/T* and *c.516T/T* genotypes are reported to be at risk of EFV plasma concentrations associated with toxicity (Lee et al., 2014). Several recent studies performed in Africa have reported a *CYP2B6 c.516T*-allele frequency ranging from 0.20 to 0.49 (Haas et al., 2004; Klein et al., 2005; Mehlotra et al., 2006; Penzak et al., 2007; Gross et al., 2008; Nyakutira et al., 2008; Mukonzo et al., 2009; Parathyras et al., 2009; Ciccacci et al., 2010; Gounden et al., 2010; Jamshidi et al., 2010; Ikediobi et al., 2011; Brown et al., 2012; Li et al., 2012; Maimbo et al., 2012; Swart et al., 2012b; Ngaimisi et al., 2013; Sarfo et al., 2014; Colic et al., 2015). These studies also reported an association of the *CYP2B6 c.516T*-allele with high EFV plasma concentrations (Mukonzo et al., 2014; Naidoo et al., 2014; Sarfo et al., 2014; Bisaso et al., 2015; Colic et al., 2015; Dhoro et al., 2015; Dickinson et al., 2015; Sinxadi et al., 2015). Thus, *CYP2B6 c.516G*>*T* genotyping has been suggested to identify poor metabolizers as part of precision medicine (Haas, 2005; Haas et al., 2005; Nolan et al., 2006; Cabrera et al., 2009; Swart et al., 2013). Another *CYP2B6* allele, *CYP2B6 c.983C* is also a predictor of deficient CYP2B6 activity associated with increased EFV plasma concentrations in certain populations (Wyen et al., 2008; Holzinger et al., 2012; Sarfo et al., 2013; Swart et al., 2013).

Focus on the pharmacogenetics of EFV has mostly been on genetic variation in *CYP2B6* with recent studies also investigating the role of other genetic variants in *CYP1A2, CYP2A6, CYP3A4, CYP3A5, UGT2B7, ATP binding cassette subfamily B member 1 (ABCB1)* and nuclear receptors pregnane X receptor (PXR, *NR1I2*) and constitutive androstane receptor (CAR, *NR1I3*) in EFV metabolism (Mukonzo et al., 2009; Burhenne et al., 2010; Elens et al., 2010; Wyen et al., 2011; Swart et al., 2012a,c; Cortes et al., 2013; Sarfo et al., 2013; Sukasem et al., 2014; Haas et al., 2014a,b). The weakness with most of the studies has been characterization of each gene in isolation, making it difficult to evaluate the contribution of each variant together with other variants that cause deficient activity. Thus, in this study we assessed the effects of other variants, including 3′-untranslated region (UTR) variation in genes coding for DMEs involved in EFV metabolism, on EFV plasma concentration. Previous studies trying to account for the genetic contribution to inter-individual differences in EFV plasma concentration were only able to explain a portion of the observed variability (Holzinger et al., 2012; Swart et al., 2013). The aim of the current study was to investigate the role of genetic variation in *CYP1A2, CYP2B6, UGT2B7*, and *NR1I2* especially polymorphisms within the 3′-UTR on EFV metabolism.

## Methods

### Study participants

Bantu-speaking South African HIV/AIDS patients (*n* = 301) were recruited from Themba Lethu Clinic, Gauteng, South Africa and were receiving EFV-based treatment for at least 6 months. The participants were unrelated adults as explained previously in Swart et al. (2013). A whole blood sample (5 mL) was obtained from each subject and used for plasma preparation (14–18 h after EFV dose) and DNA extraction as described previously by Swart et al. (2013). Ethical and study approval (HREC REF 103/2009 and M080124) was provided by the University of Cape Town Health Sciences Faculty Human Research Ethics Committee, Cape Town, South Africa, and the University of Witwatersrand Human Research Ethics Committee, Gauteng, South Africa. Written informed consent was obtained from all participants and this study was performed in accordance with guidelines of the Helsinki Declaration of 2008.

### Selection of single nucleotide polymorphisms (SNPs) and genotyping methods used

Earlier studies have mostly investigated the role of exonic and promoter single nucleotide polymorphisms (SNPs) in *CYP1A2, CYP2A6, CYP2B6*, and *CYP3A* on EFV plasma concentration (Wyen et al., 2008; Holzinger et al., 2012; Swart et al., 2012a,c, 2013; Cortes et al., 2013; Sarfo et al., 2013; Evans et al., 2015; Sinxadi et al., 2015). This study is a continuation emphasizing the role of genetic variation in the 3′-UTR of genes which products are either pharmacokinetic or pharmacodynamic targets of EFV. Thus, additional SNPs in *CYP1A2* [EMBL: ENSG00000140505; Genbank: NC_000015.10, NM_000761], *CYP2B6* [EMBL: ENSG00000197408; Genbank: NC_000019.10, NM_000767], *UGT2B7* [EMBL: ENSG00000171234; Genbank: NC_000004.12, NM_001074], and *NR1I2* [ENSG000000144852; Genbank: NC_000003.12, NM_022002] were selected for genotyping. The SNPs were selected for investigation based on functional significance. SNPs were genotyped using polymerase chain reaction-restriction fragment length polymorphism (PCR-RFLP), SNaPshot mini-sequencing or cycle sequencing. PCR-RFLP genotyping and SNaPshot mini-sequencing methods were designed and PCR amplification conditions, digestion patterns and internal SNaPshot primer sequences are shown in Supplementary Table S1.

PCR amplification for the 3′-UTR of *CYP1A2, CYP2B6*, and *NR1I2* was performed as described by Swart and Dandara (2014). PCR amplification was followed by digestion using the appropriate restriction endonuclease (Supplementary Table S1) when PCR-RFLP genotyping was used. Direct cycle sequencing of the 3′-end of *NR1I2* 3′-UTR with the reverse primer (Supplementary Table S1) was also used for further genotyping. The *CYP1A2 (c.9-154C*>*A), CYP2A6 (c.1093G*>*A), CYP2B6 (c.136A*>*G, c.485-18C*>*T, c.516G*>*T, c.785A*>*G, c.983T*>*C), CYP3A4 (c.-392G*>*A), CYP3A5 (c.219-237G*>*A, c.624G*>*A, c.1035_1036insT, c.14T*>*C), ABCB1 (c.1236T*>*C, c.193A*>*G,c.3435T*>*C, c.2677G*>*T/A), NR1I2 (c.448*+*72G*>*T, c.96-7659C*>*T, c.912-93G*>*A)*, and *NR1I3 (c.540C*>*T, c.239-1089T*>*C, c.239-99C*>*T)* SNPs were genotyped using either PCR-RFLP or SNaPshot mini-sequencing as described previously by Swart et al. (2012a), Swart et al. (2012b), Swart et al. (2012c), Swart et al. (2013), and Evans et al. (2015).

PCR amplification of *UGT2B7* was performed using the following conditions: 3 min at 94°C; 40 cycles of 94°C for 30 s, an annealing temperature of 58°C (PCR fragment including *UGT2B7 c.-161T*>*C, c.372A*>*G, c.211G*>*T*, and *c.673G*>A) or 60°C (PCR fragment including *UGT2B7 c.733A*>*G* and *c.802T*>*C*) for 30 s, 72°C for 2 min; and 10 min at 72°C for final extension (Supplementary Table S1). PCR reactions were carried out using a “T100 Thermal cycler” from Bio-Rad (Bio-Rad Laboratories, Hercules, CA, USA). Each PCR reaction contained the following reagents; 50–100 ng of genomic DNA, 1 X Green GoTaq Reaction Buffer (Promega Corporation, Madison, WI, USA), 0.2 mM of each of the deoxynucleotide triphosphates (dNTPs) (Bioline, London, UK); 1.5 mM MgCl_2_ (Promega Corporation, Madison, WI, USA); 40 pmol of the forward and reverse primers (Integrated DNA Technologies, Inc., Coralville, IA, USA); and 1 U of GoTaq DNA Polymerase (Promega Corporation, Madison, WI, USA).

For SNaPshot genotyping of four *UGT2B7* SNPs, 5 μL of each PCR product was pooled and cleaned-up using 1 U FastAP and 2 U *ExonucleaseI* (Fermentas Life Sciences, Burlington, Canada). The FastAP and *ExonucleaseI* reaction was incubated at 37°C for 1 h followed by inactivation of the enzymes at 75°C for 15 min. SNaPshot single base extension was performed on the “GeneAmp® PCR System 2700” (Applied Biosystems, Carlsbad, CA, USA) using the following conditions; 96°C for 10 s, 50°C for 5 s and 60°C for 30 s for 25 cycles. One microliter SNaPshot™ Multiplex Mix (Applied Biosystems, California, USA) and the pooled internal SNaPshot primers (Supplementary Table S1) were used in the SNaPshot reaction (10 μL) (Integrated DNA Technologies, Inc., Coralville, IA, USA). The clean-up reaction was repeated using cycling conditions as mentioned before. An ABI 3130xl Genetic Analyzer (Applied Biosystems, Carlsbad, CA, USA) was used for capillary electrophoresis and GeneMapper^©^ Software version 4.1 (Applied Biosystems, Carlsbad, CA, USA) was used to analyse results.

### Statistical analysis

SHEsis statistical program (Shi and He, 2005; Li et al., 2009) was used for linkage disequilibrium (D' and r^2^) analysis. Statistical analyses were performed using Graphpad Prism (Version 5, GraphPad Software Inc., San Diego, CA) and STATA (Version 11, StatSoft, USA) statistical programs. Mann Whitney or Kruskal-Wallis (with Dunn's Multiple Comparison) tests were used to assess association between clinical parameters, genotypes for each SNP or haplotypes and median EFV plasma concentration. EFV plasma concentrations were allocated to each of the observed haplotypes (i.e., in the event of homozygosity, the value was counted as two observations). Multivariate logistic regression analysis was performed to identify predictors of EFV plasma concentration by including covariates from the univariate analysis with a *p* ≤ 0.15, followed by stepwise backward removal. Statistical significance for the univariate analysis was defined as *p* < 0.05/N (*N* = number of SNPs or haplotypes in the analysis), while significance for the multivariate analysis was defined as *p* < 0.05/N (*N* = number of covariates in the analysis) to correct for multiple testing. Sensitivity, specificity, positive, and negative predictive values were calculated to determine the predictive value of each SNP in predicting EFV plasma concentration above 4 μg/mL.

Variant allele frequencies in the South African population group were compared to frequencies in other world populations obtained from literature. The populations included; Luhya in Webuye (Kenya), Yoruba in Ibadan (Nigeria), Utah residents with Northern, and Western European ancestry, Finnish, British, and Scottish, Iberians (Spain), Toscani (Italy), Han Chinese, Southern Han Chinese, Japanese, Colombians, Mexican ancestry from Los Angeles USA, Puerto Ricans, and Americans of African ancestry in SW USA (1000 Genomes Project and International HapMap Project). Pearson's χ^2^-test or Fisher's exact test were used to compare the allele frequencies of the South Africans to previously published frequencies in other populations.

## Results

### Basic characteristics

The majority (75%) of participants were women of median age 40 years (range: 22–75 years). Only 7% of the patients smoked tobacco and 10% consumed alcohol. EFV plasma concentrations were available for 74% (*n* = 222∕301) of the patients. Information on clinical parameters including; disease stage, BMI, CD-4 cell count and viral load were available from hospital records and were reported previously by Swart et al. (2012a), Swart et al. (2012c), and Swart et al. (2013). Adherence to ARV therapy was self-reported and four patients reported missing a day of treatment. Non-ARV medication co-administrated with EFV included vitamin B and rifampicin and 38% (*n* = 113) of HIV/AIDS patients were treated for opportunistic infections.

### Genotype frequencies and linkage disequilibrium analysis

**Figure 1:**
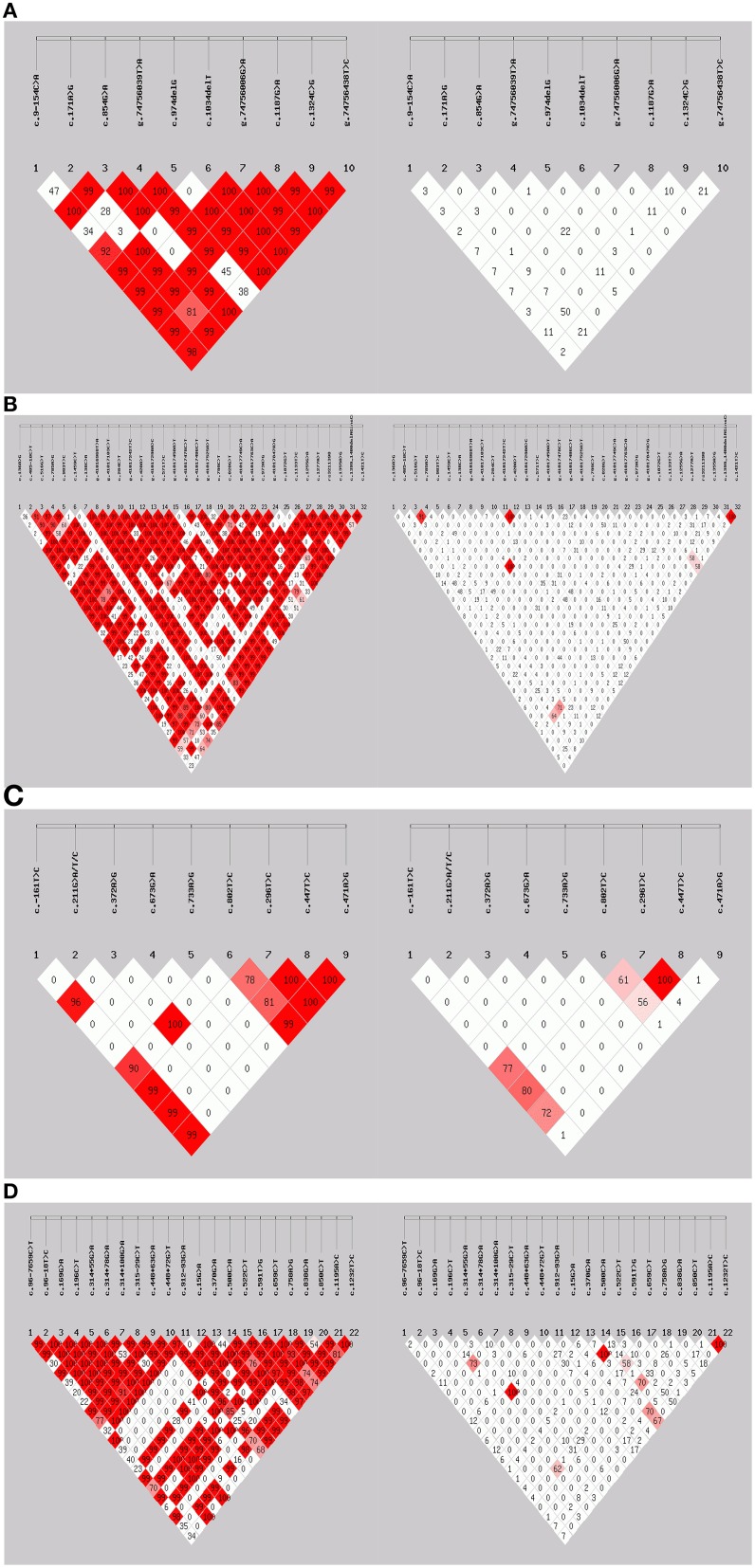
**Linkage disequilibrium analysis**. **(A)** *CYP1A2*, **(B)** *CYP2B6*, **(C)** *UGT2B7*, and **(D)** *NR1I2*. For each, the left figure represents D′-values while the right figure represents r^2^-values, respectively.

*UGT2B7 c.211G*>*T, c.673G*>*A*, and *c.733A*>*G* SNPs which have been reported in other populations (Guillemette, 2003; Zhang et al., 2008; Tian et al., 2012; Kim et al., 2014), were monomorphic in this South African group. Genotype frequencies for SNPs in the South African HIV/AIDS patients are shown in Table 1. Hardy-Weinberg equilibrium (HWE) was calculated for each SNP and only the genotype frequencies of the *UGT2B7 c.372A*>*G* SNP deviated. Linkage disequilibrium (LD) analysis was performed for all SNPs in *CYP1A2, CYP2A6, CYP2B6, CYP3A4, CYP3A5, UGT2B7, ABCB1, NR1I2*, and *NR1I3* (including SNPs genotyped previously, Swart et al., 2012a,c, 2013; Swart and Dandara, 2014; Evans et al., 2015). D′ and r^2^ (correlation coefficient preferred by the HapMap project) are both measures of linkage disequilibrium, however, these coefficients are not independent of allelic frequencies. Figures 1A–D show LD plots with D′ or r^2^ for SNP pairs in *CYP1A2, CYP2B6, UGT2B7*, and *NR1I2*. Similar to previously reported findings (Mehlotra et al., 2007; Swart et al., 2013; Haas et al., 2014a), the r^2^ LD coefficient was low for *CYP2B6 c.516G*>*T-c.983T*>*C* SNP pair (r^2^ = 0.034), whereas D′ was 0.901.

**Table 1:** **Association of basic characteristics, clinical parameters, and genetic variation in *CYP1A2, CYP2A6, CYP2B6, CYP3A4, CYP3A5, UGT2B7, ABCB1, NR1I2*, and *NR1I3* with median efavirenz plasma concentration**.

**Basic characteristics or clinical parameters**					***N***	**Median EFV conc. (μg/mL)**	***P*-value**
					222	2.56 (0.04-34.40)	
Gender	Male				54 (0.25)	2.445	0.9288
	Female				167 (0.75)	2.590	
Age					218	2.575	0.6040
Smoking	Yes				17 (0.08)	1.880	0.2877
	No				201 (0.92)	2.590	
Alcohol consumption	Yes				24 (0.11)	1.865	0.0150
	No				194 (0.89)	2.640	
BMI at baseline					192	2.615	0.0886
Disease stage	1				128 (0.59)	2.555	0.2217
	2				5 (0.02)	1.640	
	3				66 (0.31)	2.670	
	4				17 (0.08)	2.990	
Concurrent treatment of tuberculosis	Yes				14 (0.13)	3.480	0.3361
	No				90 (0.87)	2.505	
**Gene**	**SNP ID**	**SNP position**	**Genotype**	**Genotype frequency *N* (freq)**			
CYP1A2	rs762551^*^	c.9-154C>A	C/C	35 (0.12)	26	3.180	0.2734
			C/A	151 (0.53)	114	2.640	
			A/A	101 (0.35)	76	2.270	
	rs45564134	c.974delG	G/G	202 (0.77)	150	2.505	0.8789
			G/- and -/-	62 (0.23)	50	2.735	
CYP2A6	rs28399454^*^	c.1093G>A	G/G	253 (0.86)	190	2.615	0.1920
			G/A and A/A	40 (0.14)	32	2.175	
CYP2B6	rs35303484^*^	c.136A>G	A/A	248 (0.84)	187	2.540	0.7802
			A/G	33 (0.11)	27	2.660	
			G/G	14 (0.05)	8	3.095	
	rs4803419^*^	c.485-18C>T	C/C	247 (0.89)	188	2.640	0.0045
			C/T and T/T	30 (0.11)	23	1.840	
	rs3745274^*^	c.516G>T	G/G	107 (0.36)	79	1.970	<**0.0001**
			G/T	133 (0.45)	100	2.510	
			T/T	55 (0.19)	43	7.490	
	rs2279343^*^	c.785A>G	A/A	110 (0.38)	81	1.940	<**0.0001**
			A/G	132 (0.45)	100	2.510	
			G/G	51 (0.17)	40	7.695	
	rs28399499^*^	c.983T>C	T/T	253 (0.87)	192	2.395	<**0.0001**
			T/C	32 (0.11)	21	3.620	
			C/C	5 (0.02)	5	21.80	
	rs707265	c.1355A>G	A/A	15 (0.05)	14	2.590	0.7919
			A/G	75 (0.29)	54	2.630	
			G/G	172 (0.66)	130	2.520	
	rs1042389	c.1421T>C	T/T	174 (0.63)	133	2.970	0.0065
			T/C	90 (0.32)	64	2.190	
			C/C	15 (0.05)	10	2.270	
CYP3A4	rs2740574^*^	c.-392G>A	G/G	146 (0.51)	109	2.560	0.8630
			G/A	121 (0.42)	91	2.550	
			A/A	21 (0.08)	16	2.570	
CYP3A5	rs776746^*^	c.219-237G>A	G/G	7 (0.02)	5	7.020	0.1855
			G/A	77 (0.26)	58	2.805	
			A/A	211 (0.72)	159	2.400	
	rs10264272^*^	c.624G>A	G/G	195 (0.67)	148	2.590	0.5989
			G/A and A/A	96 (0.33)	72	2.515	
	rs41303343^*^	c.1035_1036insT	–/–	282 (0.96)	215	2.550	0.6810
			–/T and T/T	12 (0.04)	6	2.590	
	rs15524^*^	c.14T>C	T/T	37 (0.13)	29	2.770	0.2638
			T/C	114 (0.39)	90	2.670	
			C/C	144 (0.49)	103	2.280	
UGT2B7	rs7668258	c.-161T>C	T/T	15 (0.07)	14	2.775	0.2885
			T/C	75 (0.34)	58	2.385	
			C/C	133 (0.60)	96	2.595	
ABCB1	rs1128503^*^	c.1236T>C	T/C and T/T	60 (0.21)	42	2.730	0.1504
			C/C	235 (0.79)	180	2.470	
	rs2032582^*^	c.2677G>T/A	G/G	283 (0.96)	215	2.560	0.3387
			G/A and G/T	10 (0.04)	7	2.470	
	rs1045642^*^	c.3435T>C	T/C and T/T	62 (0.21)	43	2.470	0.6126
			C/C	233 (0.79)	179	2.560	
	rs3842^*^	c.193A>G	A/A	189 (0.64)	140	2.670	0.1378
			A/G	96 (0.33)	75	2.220	
			G/G	10 (0.03)	7	3.160	
NR1I2 (PXR)	rs2472677^*^	c.96-7659C>T	C/C	97 (0.36)	68	2.660	0.8439
			C/T	133 (0.50)	102	2.555	
			T/T	36 (0.14)	28	2.445	
	rs3732356^*^	c.448+72G>T	G/G	10 (0.03)	6	4.710	0.7447
			G/T	123 (0.42)	99	2.680	
			T/T	162 (0.55)	117	2.390	
	rs6785049^*^	c.912-93G>A	G/G	264 (0.95)	199	2.560	0.9249
			G/A and A/A	14 (0.05)	11	2.480	
	rs3732360	c.522C>T	C/C	123 (0.45)	93	2.590	0.1552
			C/T	115 (0.42)	87	2.600	
			T/T	36 (0.13)	23	2.130	
	rs1054190	c.659C>T	C/C	195 (0.90)	152	2.545	0.9843
			C/T and T/T	22 (0.10)	19	2.600	
	rs1054191	c.838G>A	G/G	195 (0.72)	152	2.655	0.1553
			G/A and A/A	77 (0.28)	55	2.380	
	rs3814057	c.1195A>C	A/A	43 (0.22)	43	2.480	0.3185
			A/C	108 (0.54)	108	2.630	
			C/C	49 (0.24)	49	2.340	
NR1I3 (CAR)	rs2307424^*^	c.540C>T	C/C	270 (0.91)	208	2.575	0.6195
			C/T	25 (0.09)	14	2.510	
	rs2502815^*^	c.239-99C>T	C/C	180 (0.61)	144	2.505	0.2412
			C/T	100 (0.34)	65	2.770	
			T/T	15 (0.05)	13	2.140	
	rs3003596^*^	c.239-1089T>C	T/T	53 (0.18)	39	2.150	0.1365
			T/C	140 (0.48)	104	2.515	
			C/C	100 (0.34)	78	2.805	

*P < 0.001 were considered significant and are shown in bold*.

* *Genotype-phenotype associations were previously reported by Swart et al. (2012a), Swart et al. (2012c), Swart et al. (2013), and Evans et al. (2015)*.

### Correlation of efavirenz plasma concentration with genetic variation and clinical parameters

The median EFV plasma concentration was 2.6 μg/mL (range: 0.04–34.4 μg/mL). A large degree of inter-individual variability (860-fold) was observed which has implications for ART response. Three percent (*n* = 7) of patients had EFV plasma concentrations below therapeutic range (1–4 μg/mL), while 31% (*n* = 69) of patients presented with EFV plasma concentrations above 4 μg/mL. Gender, age, smoking, body mass index (BMI) at baseline, disease stage, and concurrent treatment of tuberculosis (TB) did not have a significant effect on median EFV plasma concentrations, while a trend towards low EFV plasma concentration was observed for alcohol consumption (*p* = 0.015; Table 1). The genotypes of each SNP were correlated with EFV plasma concentration and data is presented for SNPs which were successfully genotyped in at least 75% of participants. As expected, high median EFV plasma concentrations were observed among carriers of the following genotypes: *CYP2B6 c.516T/T* (7.49 μg/mL), *CYP2B6 c.785G/G* (7.70 μg/mL), and *CYP2B6 c.983C/C* (21.80 μg/mL; Table 1). A trend towards low EFV plasma concentrations for *CYP2B6 c.485-18C/T* and *T/T* genotype carriers (*p* = 0.0045) and *CYP2B6 c.1421T/C* and *C/C* genotype carriers (*p* = 0.0065) was observed (Table 1).

Haplotypes were inferred for each patient by including SNPs with a variant allele frequency greater than 0.1 for *CYP1A2, CYP2B6*, and *NR1I2* (Supplementary Table S2). No significant differences in median EFV plasma concentrations were observed among haplotypes in *CYP1A2, UGT2B7*, and *NR1I2* (Supplementary Table S2).

Comparison of median EFV plasma concentrations for CYP2B6 haplotypes (*c.136A*>*G-c.516G*>*T-c.785A*>*G-c.284C*>*T-c.571T*>*C-c.799C*>*T-c.1072G*>*T-c.1277A*>*T-c.1355A*>*G-c.1399_1400delAGinsCA-c.1421T*>*C*) showed significantly higher EFV plasma concentrations for the *CYP2B6 A-T-G-T-(C or T)-C-G-(T or A)-G-AG-T* haplotype compared to the *A-G-A-T-C-C-G-A-G-AG-T* (*p* < 0.0001) and *A-G-A-T-C-C-G-A-G-CA-C* (*p* < 0.0001) haplotypes (Supplementary Table S2). The *CYP2B6 A-T-G-T-C-C-(G or T)-A-G-CA-C, A-T-G-T-C-C-G-A-A-AG-(T or C)* and *G-T-G-C-C-C-G-T-G-AG-T* haplotypes presented with EFV plasma concentrations above the recommended therapeutic range (8.55, 5.98, and 9.29 μg/mL). What is common in these haplotypes, is the *CYP2B6 c.516T*-allele further demonstrating its effect on CYP2B6 activity and, therefore, EFV plasma concentration.

Multivariate logistic regression analysis was performed to determine variables predictive of EFV plasma concentration by evaluating the effect of covariates (Table 2). Variables were prioritized for inclusion in the multivariate analysis based on having *p* < 0.15 in the univariate analysis. Stepwise removal of variables was performed to assess the effect of each covariate. Multivariate logistic regression analysis showed that *CYP2B6 c.516G*>*T* and *c.983T*>*C* SNPs were the two most significant predictors of EFV plasma concentration above 4 μg/mL (after correction for multiple testing with *p* < 0.006). The *NR1I2 c.239-1089T*>*C* SNP played a minor role (*p* = 0.011) in explaining variability in EFV plasma concentrations (Table 2).

**Table 2:** **Multivariate logistic regression analysis of efavirenz plasma concentrations above or below 4 μg/mL**.

**Independent variables**	**Regression coefficient**	**95% Confidence interval**	*P*-value^*^	**Change in log likelihood**	**Change in pseudo R^2^ (%)**
CYP2B6 c.516G>T	2.65	(1.75 to 3.55)	<**0.0001**	39.66	29.14
CYP2B6 c.983T>C	2.97	(1.67 to 4.23)	<**0.0001**	20.76	14.36
CYP2B6 c.485-18C>T	0.03	(−1.70 to 1.77)	0.970	1.08	0.07
CYP2B6 c.1421T>C	−0.65	(−1.51 to 0.22)	0.142	7.35	2.06
Alcohol consumption	−1.13	(−2.49 to 0.22)	0.101	1.22	0.22
BMI	−0.04	(−0.13 to 0.06)	0.444	11.71	4.07
NR1I3 c.239-1089T>C	0.89	(0.21 to 1.57)	0.011	2.44	1.62
ABCB1 c.193A>G	−0.86	(−1.66 to −0.07)	0.034	1.26	0.98

*Variables were included in the multivariate logistic regression analysis based on having a p ≤ 0.15 in the univariate analysis*.

* *P-values shown are for each variable included in the multivariate logistic regression analysis (log likelihood of -65.24 and pseudo R^2^ of 0.4265) before stepwise removal*.

*Variables remaining significant after correction for multiple testing (P < 0.006) are shown in bold*.

Expanding our investigation to find genetic variants that are important in predicting EFV plasma concentration, was motivated by the need to come up with a test for EFV dosing. Thus, sensitivity, specificity, positive predictive value (PPV), and negative predictive value (NPV) were calculated for individual SNP genotypes and combinations, considering SNPs included in multivariate logistic regression analysis. Genotypes and their combinations were used to predict EFV plasma concentrations above 4 μg/mL (Table 3 and Supplementary Table S3). Seventy-seven percent (*n* = 33∕43) of patients with the *CYP2B6 c.516T/T* genotype had EFV plasma concentrations above 4 μg/mL (Supplementary Table S3). Thus, the *CYP2B6 c.516T/T* genotype was associated with the following parameters; sensitivity of 48% and specificity of 94% in predicting EFV plasma concentrations > 4 μg/mL. Sensitivity of the *CYP2B6 c.983C/C* genotype (7%) in predicting EFV plasma concentrations > 4 μg/mL was low, but the specificity (100%) was high. Sensitivity and NPV of the *CYP2B6 c.485-18C/C* and *c.1421T/T* genotypes were high, but the specificity and PPV were low. Using the *CYP2B6 c.516G*>*T* and *c.983T*>*C* SNPs to come up with the CYP2B6 poor metabolizer phenotype (*CYP2B6 c.516T/T* or *CYP2B6 c.983C/C* or *CYP2B6 c.516G/T*+*CYP2B6 c.983T/C*) showed the best predictive model with 68% sensitivity, 93% specificity, 83% PPV, and 87% NPV, respectively (Table 3).

**Table 3:** **Validity of genotypes in predicting efavirenz plasma concentrations above 4 μg/mL**.

**Risk factors for EFV plasma concentration > 4 μg/mL**	**Sensitivity (%)**	**Specificity (%)**	**Positive predictive value (%)**	**Negative predictive value (%)**
CYP2B6 c.516T/T	47.8	93.5	76.7	79.9
CYP2B6 c.983C/C	7.2	100	100	70.0
CYP2B6 c.485-18C/C	97.1	14.7	35.1	91.3
CYP2B6 c.1421T/T	83.1	44.4	40.6	85.1
NR1I3 c.239-1089C/C	42.0	67.8	37.2	72.0
ABCB1 c.193G/G	4.3	97.4	42.9	69.3
CYP2B6 poor metabolizers (i.e., c.516T/T or c.983C/C or c.516G/T + c.983T/C)	68.1	93.4	82.5	86.5
CYP2B6 poor metabolizers together with CYP2B6 c.485-18C carriers	69.1	93.0	82.5	86.3
CYP2B6 poor metabolizers together with CYP2B6 c.1421T carriers	66.2	94.4	84.3	85.9
CYP2B6 poor metabolizers together with NR1I3 c.239-1089C carriers	58.0	97.3	90.9	83.2
CYP2B6 poor metabolizers together with ABCB1 c.193G carriers	17.4	97.4	75.0	72.1

### Comparison of allele frequencies between populations

Allele frequencies of SNPs for other world ethnic groups were obtained from the National Centre for Biotechnology Information (NCBI) dbSNP database (http://www.ncbi.nlm.nih.gov/) and the 1000 Genomes Project (http://www.1000genomes.org/). Statistically significant differences were clearly observed when the South African group was compared to American, Asian, and European groups. In addition, differences were also observed when the South African group was compared to the Yoruba and Luhya (Table 4). For example, *NR1I2 c.659T*-allele was present at a frequency of 0.05 in the South African group, absent among Asians, yet comparable to that of the Americans (0.07–0.13). In contrast, the *UGT2B7 c.211T*-allele was absent among South Africans, other Africans, and Europeans, but present among Asians (0.11–0.14). Comparison of the *NR1I2 c.758G*-allele showed a significantly higher frequency among the Yoruba (0.19), the Luhya (0.10), and African Americans (0.11) compared to a frequency of 0.06 among South Africans.

**Table 4:** **Variant allele frequencies in the South African population compared to other world populations**.

**Gene**	**SNP position**	**Variant allele**	**South Africans (This study, *n* = 222)**	**African populations**		**European populations**					**East Asian populations**			**American populations**			
				**LWK (*n* = 97)**	**YRI (*n* = 88)**	**CEU (*n* = 87)**	**FIN (*n* = 93)**	**GBR (*n* = 88)**	**IBS (*n* = 14)**	**TSI (*n* = 98)**	**CHB (*n* = 97)**	**CHS (*n* = 101)**	**JPT (*n* = 89)**	**CLM (*n* = 61)**	**MXL (*n* = 66)**	**PUR (*n* = 55)**	**ASW (*n* = 61)**
CYP2B6	c.1355A>G^*^	A	0.20	0.16	0.18	**0.40**	**0.43**	**0.39**	0.29	**0.36**	0.33	**0.37**	**0.34**	0.17	0.23	0.25	0.21
	c.1421T>C^*^	C	0.22	0.35	0.24	0.19	0.18	0.25	0.36	0.18	0.31	0.25	0.28	0.14	0.15	0.19	0.20
UGT2B7	c.-161T>C	C	0.74	0.75	0.81	**0.52**	**0.55**	**0.47**	0.54	**0.48**	0.69	0.75	0.70	0.57	0.73	0.60	0.74
	c.211G>T	T	0.00	0.00	0.00	0.00	**0.03**	0.00	0.00	0.00	**0.14**	**0.11**	**0.14**	0.02	0.01	0.01	0.01
	c.372A>G	G	0.01	**0.11**	0.04	**0.22**	**0.20**	**0.19**	0.07	**0.18**	**0.16**	**0.16**	**0.15**	**0.12**	0.06	**0.12**	**0.07**
	c.802T>C	C	0.73	0.71	0.79	**0.50**	**0.51**	**0.40**	**0.32**	**0.47**	0.61	0.73	0.67	**0.53**	0.69	0.59	0.68
NR1I2 (PXR)	c.522C>T^*^	T	0.34	0.37	0.24	**0.80**	**0.70**	**0.75**	**0.82**	**0.76**	0.49	0.38	**0.52**	**0.73**	**0.74**	**0.73**	0.43
	c.659C>T^*^	T	0.05	0.05	0.02	**0.15**	0.05	**0.16**	0.07	**0.18**	0.00	0.00	0.00	0.13	0.08	0.10	0.07
	c.758A>G	G	0.06	0.10	**0.19**	0.04	0.07	0.05	0.00	0.02	0.05	0.07	0.04	0.07	0.01	0.04	0.11
	c.838G>A^*^	A	0.15	0.22	0.10	0.19	0.06	0.19	0.11	0.21	**0.00**	**0.01**	**0.00**	0.18	0.13	0.14	0.17
	c.1195A>C^*^	C	0.51	0.47	0.51	**0.11**	**0.21**	**0.18**	0.14	**0.18**	0.46	0.54	0.44	**0.18**	**0.20**	**0.21**	0.42
	c.1232T>C^*^	C	0.52	0.47	0.51	**0.11**	**0.21**	**0.18**	0.14	**0.18**	0.46	0.54	0.44	**0.17**	**0.20**	**0.21**	0.42

*South Africans (this study) were compared to all other world populations and only P < 0.004 were considered significant and are shown in bold*.

* *Variant allele frequencies were reported previously by Swart and Dandara (2014)*.

*LWK, Luhya in Webuye (Kenya); YRI, Yoruba in Ibadan (Nigeria); CEU, Utah residents with Northern and Western European ancestry; FIN, Finnish; GBR, British and Scottish; IBS, Iberians (Spain); TSI, Toscani (Italy); CHB, Han Chinese; CHS, Southern Han Chinese; JPT, Japanese; CLM, Colombians; MXL, Mexican ancestry from Los Angeles USA; PUR, Puerto Ricans; ASW, Americans of African ancestry in SW USA*.

## Discussion

### Implications for biomarker identification, disease diagnosis, or drug treatment

This study has taken investigation into pharmacogenetics determinants of EFV plasma concentration further by evaluating the contribution of 30 SNPs, especially those in the 3′-UTR of genes that code for DMEs metabolizing EFV. Most studies have concentrated on the role of *CYP2B6 c.516G*>*T* and *c.983T*>*C* SNPs in predicting EFV plasma concentration. We report on large inter-individual variability in EFV plasma concentration supporting previous studies among South Africans (Gounden et al., 2010) and Swiss patients (Marzolini et al., 2001; Colombo et al., 2006). Thirty-four percent of the patients presented with EFV plasma concentrations outside the therapeutic range (3% below and 31% above) and are at risk of failing therapy or presenting with ADRs, respectively. This is similar to observations by Marzolini et al. (2001), Ståhle et al. (2004), Lubomirov et al. (2011), Mukonzo et al. (2013), and Sanchez Martin et al. (2013). Poor virologic suppression and ADRs lead to patient's health deteriorating which could lead to increased non-adherence to treatment (ter Heine et al., 2008).

Drug response is a complex phenotype and multiple factors including ethnicity, sex, age, body weight, drug-drug, and drug-food interactions, hepatic impairment, disease state, pregnancy, and host genetic variation can influence the pharmacokinetic variability of EFV (Burger et al., 2006; Rotger et al., 2006; Stohr et al., 2008). Alcohol consumption and BMI were included in the multivariate logistic regression analysis as potential contributors to EFV plasma concentrations > 4 μg/mL. However, the majority of HIV/AIDS patients were females which might be the reason for the very low smoking and alcohol consumption among the patients. Smoking and alcohol consumption is generally a less common lifestyle among female Bantu-speaking South Africans, compared to other racial groups. In this study, EFV plasma concentrations were higher in patients with a lower BMI supporting observations by Poeta et al. (2011) and Stohr et al. (2008).

Genetic variation in genes coding for DMEs responsible for EFV metabolism is known to affect EFV plasma concentrations, thus, a comprehensive analysis on the contribution of genetic variants in genes coding for enzymes involved in EFV metabolism was carried out. *CYP2B6 c.516G*>*T* and *c.983T*>*C* SNPs are well-characterized predictors of EFV plasma concentration (Haas et al., 2004; Swart et al., 2013) and here we re-confirm their importance. Dhoro et al. (2015) reported that Zimbabwean HIV/AIDS patients carrying the *CYP2B6 c.516T/T* genotype had 63% decreased EFV clearance. Mukonzo et al. (2014) also reported an association of *CYP2B6 c.516T/T* genotype with increased EFV exposure among Ugandan patients leading to their recommendation of daily doses of 300 mg for *CYP2B6 c.516T/T* genotype carriers.

Five individuals with the *CYP2B6 c.983C/C* genotype were identified in this study and four of these patients had nearly five-fold higher EFV plasma concentrations (34.4, 21.8, 12.1, and 22.3 μg/mL) with respect to the therapeutic range upper limit of 4 μg/mL. *CYP2B6 c.983C*-allele has been associated with significant differences in EFV exposure among individuals of African and Caucasian ancestry (Gatanaga et al., 2007; Wyen et al., 2008; Haas et al., 2009). Multivariate logistic regression analysis showed an additive effect between the *CYP2B6 c.516G*>*T* and *c.983T*>*C* SNPs with an almost two-fold higher EFV plasma concentration, a finding in agreement with reports by Wyen et al. (2008) and Schipani et al. (2011). These observations suggest that *CYP2B6* polymorphisms can be used as biomarkers of EFV plasma concentration, further arguing for genotype-assisted dosing with respect to the use of EFV-containing HAART (Rotger et al., 2007; Rotger and Telenti, 2008; Schipani et al., 2011). Recommendations based on pharmacogenetics knowledge are being proposed, such as gradual reductions in EFV dose from 600 to 400 or 200 mg/day for intermediate metabolizer and poor metabolizer patient groups, respectively (Gatanaga et al., 2007; Cabrera et al., 2009; Mukonzo et al., 2014). The recently completed ENCORE1 study (including 37% African individuals) concluded that HIV suppression was comparable between EFV doses of 400 and 600 mg daily (Dickinson et al., 2015). Bisaso et al. (2015) suggested a 7% reduction in neuropsychological impairment probability at an EFV dose of 450 mg/day. The cost-effectiveness of using *CYP2B6* genotyping to adjust EFV dosage was assessed recently by Schackman et al. (2015). The cost-effectiveness ratio of genotype-assisted dosing compared to a lower universal dose remains greater than $ 100,000/quality-adjusted life years, unless HIV RNA suppression is decreased by more than 15% (Schackman et al., 2015).

In addition to univariate analysis, haplotypes were inferred for each HIV/AIDS patient and median EFV plasma concentrations were compared between haplotype groups. The effect of the *CYP2B6 c.516G*>*T* SNP is shown by the significantly higher median EFV plasma concentration for the *CYP2B6 A-T-G-T-(C or T)-C-G-(T or A)-G-AG-T* haplotype compared to the *A-G-A-T-C-C-G-A-G-AG-T* and *A-G-A-T-C-C-G-A-G-CA-C* haplotypes. However, only four of the nine haplotypes with the *CYP2B6 c.516T*-allele, had median EFV plasma concentrations above 4 μg/mL. This observation points to the potential involvement of additional SNPs in *CYP2B6* or other genes coding for DMEs that play a role in EFV metabolism.

Furthermore, multivariate logistic regression analysis was performed to identify additional contributors to variability in EFV plasma concentration. Few studies have investigated the effect of SNPs in the 3′-UTR of genes coding for DMEs in drug response. A trend towards low EFV plasma concentration was observed for the *CYP2B6 c.1421C*-allele which is located in the 3′-UTR. The *CYP2B6 c.1421T*>*C* SNP is predicted to affect the binding of miR-3612, miR-650, miR-4266, miR-4779, miR-4695-5p, miR-765, miR-4476, miR-6077, miR-6876-5p, and miR-8065 (Swart and Dandara, 2014) and, thus, potentially alter levels of CYP2B6 mRNA.

In addition to *CYP2B6 c.516G*>*T* and *c.983T*>*C* SNPs, the *NR1I3 c.239-1089T*>*C, ABCB1 c.193A*>*G, CYP2B6 c.485-18C*>*T*, and *CYP2B6 c.1421T*>*C* SNPs potentially further contribute to variability in EFV plasma concentration. However, sensitivity, specificity, positive, and negative predictive values for each SNP alone was low, but combining the *CYP2B6 c.516G*>*T* and *c.983T*>*C* SNPs improved the predictive values further arguing for their utility in a pharmacogenetics-based test. Further, addition of the *CYP2B6 c.485-18C*>*T* SNP improved the sensitivity from 68 to 69%. Inclusion of the *NR1I3 c.239-1089T*>*C, ABCB1 c.193A*>*G*, and *CYP2B6 c.1421T*>*C* SNPs did not improve predictive ability of the CYP2B6 poor metabolizers (PMs) genotyping test.

### Comparison of allele frequencies

The current study shows major differences in variant allele frequencies of SNPs between ethnic groups. Allele frequencies were significantly different between African and other populations especially for *NR1I2 c.522C*>*T, NR1I2 c.1195A*>*C*, and *NR1I2 c.1232T*>*C* SNPs. *NR1I2* codes for the nuclear receptor PXR and is responsible for regulating most genes coding for Phase I, Phase II enzymes, and transporters. PXR is activated by many ligands including the antimalarial drug artemisinin (Burk et al., 2005) and EFV (Healan-Greenberg et al., 2008). Furthermore, it appears the *UGT2B7 c.211G*>*T* SNP is specific to Asian populations, while *NR1I2 c.659C*>*T* and *c.838G*>*A* SNPs are rare among Asians. Differences in allele distribution are likely to contribute to the observed ethnic variability in drug levels.

Inter-ethnic variations in drug exposure may possibly result in varying clinical treatment outcome or adverse event profiles between populations (Ngaimisi et al., 2013). African populations are the most genetically diverse, thus, should be utilized in clinical trials to enable the teasing out of genetic correlates of drug response. The high prevalence of HIV/AIDS in South Africa, combined with the exposure to ARVs necessitates characterizing the genetic variation of pharmacogenetically relevant genes to improve drug response (Colic et al., 2015).

The current study is a continuation of earlier studies to assess the contribution of 30 SNPs, especially those in the 3′-UTR of genes that code for DMEs metabolizing EFV. The association or trend toward low median EFV plasma concentration observed for the *NR1I3 c.239-1089T*-allele, the *ABCB1 c.193A*-allele, the *CYP2B6 c.485-18T*-allele, and the *CYP2B6 c.1421C*-allele need to be investigated in further replication and larger cohorts. Compared to *CYP2B6 c.516G*>*T* and *c.983T*>*C* SNPs, the *NR1I3 c.239-1089T*>*C, ABCB1 c.193A*>*G, CYP2B6 c.485-18C*>*T*, and *CYP2B6 c.1421T*>*C* SNPs seem to play minor roles in predicting EFV plasma concentrations above 4 μg/mL.

## Author contributions

CD conceived of the study, designed, coordinated the study, recruited all the participants, and did all the sampling, also assisted with statistical data analysis, helped to draft the manuscript and approved the final version. MSW, JE, MSK carried out the molecular genetic characterization experiments and MSW drafted the manuscript. SC, LW, and PS carried out the LC/MS/MS analysis of EFV concentration. All authors read and approved the final manuscript.

## Funding

Research reported in this publication was supported by the South African Medical Research Council (SAMRC) Self-Initiated Research Grant awarded to CD for pharmacogenetics research. MSW was awarded study bursaries by the SAMRC and University of Cape Town Carnegie Corporation *Developing the Next Generation of Academics* Program (infectious diseases research focus). The views and opinions expressed are not those of the SAMRC but of the authors of the material published.

### Conflict of interest statement

The authors declare that the research was conducted in the absence of any commercial or financial relationships that could be construed as a potential conflict of interest.
