# Supplementary material for: An Expanded Analysis of Pharmacogenetics Determinants of Efavirenz Response that Includes 3′-UTR Single Nucleotide Polymorphisms among Black South African HIV/AIDS Patients
Source: Front Genet. 2016 Jan 7;6:356. doi: 10.3389/fgene.2015.00356 (PMC4703773; doi:10.3389/fgene.2015.00356)
Supplement: Supplementary file 1 [file Table1.docx]

**Supplementary Table S1: PCR amplification conditions and PCR-RFLP genotyping or SNaPshot mini-sequencing**

| **Gene** | **SNP ID** | **SNP position** | **Primer sequence (5'-3')** | **Annealing temp** | **PCR product size (bp)** | **Method of detection** |
| --- | --- | --- | --- | --- | --- | --- |
| CYP1A2 | rs45564134 | c.974delG | F:CGCAGGTTCAAGCAATCC & R:AGGACTCAAGCACCAAGAGC | 61°C | 1161 | PCR-RFLP: *SsiI*  G:44,159,258,336,364 & delG:203,258,336,364 |
| CYP2B6 | rs707265 | c.1355A>G | F:GTGGTGCCATCTCTGTCCA & R:AGAGTTGGCATTGAGGTGAGAG | 61°C | 981 | PCR-RFLP: *NlaIII*  G:10,36,113,322,475 & A:10,25,36,67,113,475 |
|  | rs1042389 | c.1421T>C | F:GTGGTGCCATCTCTGTCCA & R:AGAGTTGGCATTGAGGTGAGAG | 61°C | 981 | PCR-RFLP: *EarI*  T:255,726 & C:981 |
| UGT2B7 | rs7668258 | c.-161T>C | F:CTGATTGTTATGGTAGATGC & R:TAAGTTAGAGCTTCATGTTACTG  Internal SNaPshot Primer: TGTCAACAGTTCATTTACCTTCATTTGTCTC | 58°C | 1264 | SNaPshot |
|  | rs12233719 | c.211G>T | F:CTGATTGTTATGGTAGATGC & R:TAAGTTAGAGCTTCATGTTACTG  Internal SNaPshot Primer: ATTCTACAACTCAGATTTAGTATAAGATGTGGGATAACTTTCTATTTTAAGAG | 58°C | 1264 | SNaPshot |
|  | rs28365063 | c.372A>G | F:CTGATTGTTATGGTAGATGC & R:TAAGTTAGAGCTTCATGTTACTG  Internal SNaPshot Primer: TGTCTTATTTGTCTTATTTGATACTACATCTTTACAGAACTT | 58°C | 1264 | SNaPshot |
|  | rs146308452 | c.673G>A | F:CTGATTGTTATGGTAGATGC & R:TAAGTTAGAGCTTCATGTTACTG | 58°C | 1264 | PCR-RFLP: *TaqI*  G:91,1173 & A:1264 |
|  | rs139488772 | c.733A>G | F:TGAAACTATGTCTCTTTATTAGAAC & R:GTGTGATGGTATTAACGTG  Internal SNaPshot Primer: TTTTTATTCACCTTTTTTTTTTCTATTATGCAGTAAGACCCACT | 60°C | 978 | SNaPshot |
|  | rs7439366 | c.802T>C | F:TGAAACTATGTCTCTTTATTAGAAC & R:GTGTGATGGTATTAACGTG | 60°C | 978 | PCR-RFLP: *BtsCI*  T:347,631 & C:111,236,631 |
| NR1I2 (PXR) | rs3732360 | c.522C>T | F:CCAGGACATACACCCCTTG & R:TATTTCCACACCCCCACATT | 60°C | 1394 | PCR-RFLP: *HhaI*  C:587,807 & T:1394 |
|  | rs1054190 | c.659C>T | F:CCAGGACATACACCCCTTG & R:TATTTCCACACCCCCACATT | 60°C | 1394 | PCR-RFLP: *TspRI* C:153,209,483,604 & T:153,185,209,419,483 |
|  | rs1054191 | c.838G>A | F:CCAGGACATACACCCCTTG & R:TATTTCCACACCCCCACATT | 60°C, 2min | 1394 | PCR-RFLP: *SsiI*  G:14,27,55,79,494,780 & A:14,27,55,79,1274 |

**Supplementary Table S2: Association of haplotypes in *CYP1A2, CYP2B6, UGT2B7* and *NR1I2* with efavirenz plasma concentrations**

| **Gene** | **SNPs considered** | **Haplotype** | **Number of observations** | **Median EFV plasma concentration (µg/mL)** | **General P-value** |
| --- | --- | --- | --- | --- | --- |
| CYP1A2 | c.9-154C>A - c.171A>G - c.974delG - c.1324C>G | C-(A or G)-G-C | 171 | 2.680 (0.58-34.4) | 0.4992 |
|  |  | A-(A or G)-G-(C or G) | 211 | 2.410 (0.04-34.4) |  |
|  |  | (C or A)-(A or G)-delG-C | 54 | 2.680 (0.58-21.8) |  |
|  |  | A-G-G-G | 8 | 3.220 (1.19-5.28) |  |
| CYP2B6 | c.136A>G-c.516G>T-c.785A>G-c.284C>T-c.571T>C-c.799C>T-c.1072G>T-c.1277A>T-c.1355A>G-c.1399_1400delAGinsCA-c.1421T>C | A-G-A-C-C-(C or A)-G-(A or T)-G-AG-T | 4 | 3.220 (1.05-3.58) | **< 0.001** |
|  |  | A-G-A-T-C-C-G-A-G-AG-T | 65 | 1.910 (0.04-21.8) |  |
|  |  | A-G-A-T-C-C-G-A-G-CA-C | 70 | 2.135 (0.79-16.1) |  |
|  |  | A-G-A-T-T-C-G-A-G-AG-T | 23 | 2.250 (0.99-34.4) |  |
|  |  | A-G-A-T-T-C-G-A-A-AG-T | 73 | 2.460 (0.58-22.3) |  |
|  |  | A-G-A-T-T-C-G-A-A-AG-C | 2 | 1.955 (1.28-2.63) |  |
|  |  | A-T-A-T-C-C-T-A-G-CA-C | 3 | 0.750 (0.75-0.84) |  |
|  |  | A-T-G-C-C-A-G-T-G-AG-T | 3 | 1.600 (1.05-3.39) |  |
|  |  | A-T-(A or G)-T-C-C-G-A-G-AG-T | 2 | 2.290 (1.81-2.77) |  |
|  |  | A-T-G-T-C-C-(G or T)-A-G-CA-C | 7 | 8.550 (0.84-14.5) |  |
|  |  | A-T-G-T-C-C-G-A-A-AG-(T or C) | 6 | 5.980 (2.54-20.1) |  |
|  |  | A-T-G-T-(C or T)-C-G-(T or A)-G-AG-T | 137 | 4.690 (0.04-21.0) |  |
|  |  | G-G-(A or G)-(C or T)-C-C-G-(A or T)-G-AG-T | 4 | 2.195 (0.59-16.1) |  |
|  |  | G-G-A-T-C-C-G-A-G-AG-T | 8 | 2.165 (0.58-3.44) |  |
|  |  | G-G-A-T-C-C-G-A-G-CA-C | 4 | 3.915 (1.53-4.74) |  |
|  |  | (A or G)-(G or T)- A-T-T-C-G-A-(A or G)-AG-T | 4 | 2.255 (0.59-4.99) |  |
|  |  | G-T-G-C-C-C-G-T-G-AG-T | 4 | 9.290 (1.48-16.7) |  |
|  |  | G-T-G-C-C-A-G-T-G-AG-T | 2 | 1.475 (1.19-1.76) |  |
|  |  | G-T-G-T-C-C-G-T-G-AG-T | 18 | 2.970 (0.99-16.7) |  |
| UGT2B7 | c.-161T>C-c.211G>A/T/C-c.372A>G-c.673G>A-c.733A>G-c.802T>C-c.296T>C-c.447T>C-c.471A>G | C-G-A-G-A-C-C-C-(A or G) | 323 | 2.560 (0.04-34.4) | 0.7675 |
|  |  | C-G-A-G-A-(T or C)-T-(T or C)-A | 5 | 2.770 (1.58-5.28) |  |
|  |  | C-G-G-G-A-C-C-C-A | 5 | 1.640 (1.29-12.9) |  |
|  |  | (T or C)-G-A-G-A-T-C-C-A | 4 | 2.035 (1.19-5.85) |  |
|  |  | T-G-A-G-A-T-T-T-A | 105 | 2.560 (0.75-17.6) |  |
| NR1I2 (PXR) | c.96-7659C>T-c.314+78G>A-c.315-29C>T-c.448+72G>T-c.370G>A-c.500C>A-c.522C>T-c.838G>A-c.1195A>C-c.1232T>C | T-C-C-T-G-C-C-G-C-C | 4 | 2.840 (1.58-3.44) | 0.5340 |
|  |  | T-C-T-T-G-C-C-G-C-C | 2 | 7.555 (3.01-12.1) |  |
|  |  | T-T-(C or T)-T-G-C-C-G-C-C | 130 | 2.510 (0.58-34.4) |  |
|  |  | T-T-C-T-A-C-T-G-A-T | 7 | 1.990 (1.19-16.1) |  |
|  |  | T-T-C-T-A-C-T-A-A-T | 3 | 4.190 (1.57-4.74) |  |
|  |  | T-(C or T)-C-T-A-A-T-A-A-T | 24 | 2.285 (1.28-21.8) |  |
|  |  | T-T-C-C-G-C-C-G-A-T | 2 | 1.590 (1.30-1.88) |  |
|  |  | T-T-C-C-A-C-T-G-A-T | 3 | 2.480 (1.30-3.69) |  |
|  |  | C-C-C-T-G-C-C-G-A-T | 12 | 2.530 (2.04-13.6) |  |
|  |  | C-C-C-T-G-C-C-G-C-C | 79 | 2.250 (0.04-34.4) |  |
|  |  | C-C-C-T-A-C-T-G-(A or C)-(T or C) | 38 | 2.775 (0.99-17.6) |  |
|  |  | C-(T or C)-(C or T)-T-A-A-T-A-A-T | 20 | 2.430 (0.75-20.1) |  |
|  |  | C-C-C-C-G-C-C-G-A-T | 48 | 3.210 (1.25-34.1) |  |
|  |  | (C or T)-C-C-C-G-C-C-G-C-C | 10 | 6.120 (0.79-20.2) |  |
|  |  | C-C-C-C-A-C-C-G-A-T | 6 | 1.735 (1.29-8.26) |  |
|  |  | C-C-C-C-A-C-T-G-A-T | 32 | 2.270 (0.04-22.3) |  |
|  |  | C-C-C-C-A-A-T-A-A-T | 11 | 2.380 (1.07-7.59) |  |
|  |  | C-C-T-T-(G or A)-C-C-G-A-T | 2 | 8.115 (2.63-13.6) |  |
|  |  | C-C-T-T-G-C-C-G-C-C | 3 | 3.010 (2.63-12.1) |  |
|  |  | C-T-C-T-G-C-C-G-C-C | 3 | 2.560 (1.46-3.62) |  |
|  |  | C-T-C-T-A-C-T-G-A-T | 3 | 2.770 (1.19-13.9) |  |

Only SNPs with a variant allele frequency > 0.1 was included in the haplotype analysis for *CYP1A2, CYP2B6* and *NR1I2*. P-values < 0.001 were considered significant and are shown in bold.

**Supplementary Table S3: Number of HIV/AIDS patients with efavirenz plasma concentrations ≤ 4 µg/mL and > 4 µg/mL**

| **Genotypes/Phenotypes (and combinations)** | **Patients with EFV plasma conc. > 4 µg/mL (n = 69)** | **Patients with EFV plasma conc. ≤ 4 µg/mL (n = 153)** |
| --- | --- | --- |
| CYP2B6 c.516G/G | 9 (0.13) | 70 (0.46) |
| CYP2B6 c.516G/T | 27 (0.39) | 73 (0.48) |
| CYP2B6 c.516T/T | 33 (0.48) | 10 (0.06) |
| CYP2B6 c.983T/T | 54 (0.78) | 138 (0.93) |
| CYP2B6 c.983T/C | 10 (0.14) | 11 (0.07) |
| CYP2B6 c.983C/C | 5 (0.08) | 0 (0.00) |
| CYP2B6 c.485-18C/C | 66 (0.97) | 122 (0.85) |
| CYP2B6 c.485-18C/T | 2 (0.03) | 19 (0.13) |
| CYP2B6 c.485-18T/T | 0 (0.00) | 2 (0.02) |
| CYP2B6 c.1421T/T | 54 (0.83) | 79 (0.56) |
| CYP2B6 c.1421T/C | 10 (0.15) | 54 (0.38) |
| CYP2B6 c.1421C/C | 1 (0.02) | 9 (0.06) |
| NR1I3 c.239-1089T/T | 9 (0.13) | 30 (0.20) |
| NR1I3 c.239-1089T/C | 31 (0.45) | 73 (0.48) |
| NR1I3 c.239-1089C/C | 29 (0.42) | 49 (0.32) |
| ABCB1 c.193A/A | 52 (0.75) | 88 (0.58) |
| ABCB1 c.193A/G | 14 (0.20) | 61 (0.40) |
| ABCB1 c.193G/G | 3 (0.05) | 4 (0.02) |
| CYP2B6 PM | 47 (0.68) | 10 (0.07) |
| CYP2B6 EM | 22 0.32) | 141 (0.93) |
| CYP2B6 PM + CYP2B6 c.485-18C | 47 (0.69) | 10 (0.07) |
| CYP2B6 EM + CYP2B6 c.485-18T/T | 21 (0.31) | 132 (0.93) |
| CYP2B6 PM + CYP2B6 c.1421T | 43 (0.66) | 8 (0.06) |
| CYP2B6 EM + CYP2B6 c.1421C/C | 22 (0.34) | 134 (0.94) |
| CYP2B6 PM + NR1I3 c.239-1089C | 40 (0.58) | 4 (0.03) |
| CYP2B6 EM + NR1I3 c.239-1089T/T | 29 (0.42) | 144 (0.97) |
| CYP2B6 PM + ABCB1 c.193G | 12 (0.17) | 4 (0.03) |
| CYP2B6 EM + ABCB1 c.193A/A | 57 (0.83) | 147 (0.97) |

Note: PM=poor metaboliser; EM=extensive metaboliser, CYP2B6 PM refers to carriers of CYP2B6 c.516T/T or c.983C/C or c.516G/T + c.983T/C genotypes and CYP2B6 EM refers to carriers of CYP2B6 c.516G/G + c.983T/T or c.516G/G + c.983T/C or c.516G/T + c.983T/T genotypes
